# Plasma circulating microRNAs and symptoms of depression: Results from a population‐based study

**DOI:** 10.1111/pcn.13869

**Published:** 2025-07-14

**Authors:** Midas M. Kuilman, Tim Finke, M. Arfan Ikram, Annemarie I. Luik, Alexander Neumann, Charlotte A.M. Cecil, Mohsen Ghanbari

**Affiliations:** ^1^ Department of Epidemiology Erasmus University Medical Center Rotterdam The Netherlands; ^2^ Department of Child and Adolescent Psychiatry Erasmus University Medical Center Rotterdam The Netherlands; ^3^ Trimbos Institute – Netherlands Institute for Mental Health and Addiction Utrecht The Netherlands

**Keywords:** biomarkers, depression, MicroRNAs, population‐based study

## Abstract

**Background:**

Depression is a complex mental disorder with a multifactorial etiology. Recent research has highlighted the potential of microRNAs (miRNAs) as novel biomarkers and their involvement in the molecular pathways underlying depression; yet, these studies often focus on limited clinical samples and specific subsets of miRNAs. Here we aimed to explore plasma miRNA expression profiles associated with depressive symptoms in a population‐based study of middle‐aged and older adults.

**Methods:**

We analyzed the levels of 591 circulating miRNAs well‐expressed in plasma samples of 2,703 participants of the Rotterdam Study. The Center for Epidemiologic Studies Depression Scale was used to assess depressive symptoms in these participants. Negative‐binomial regression models were employed to explore the relationship between individual miRNA levels and depressive symptoms, adjusting for potential confounders including age, sex, BMI, and other factors.

**Results:**

Our analysis suggests 38 circulating miRNAs to be potentially associated with depressive symptoms (*P* < 0.05). Although these miRNAs did not survive multiple testing correction, our subsequent *in silico* analysis of their target genes suggests involvement in neural and psychiatric pathways, as well as enrichment for associations with depressive symptoms based on previous genome‐wide association studies.

**Conclusions:**

This study proposes several circulating miRNAs that may be associated with depressive symptoms within the general population. Our follow‐up analyses indicate that the miRNAs with the largest effect estimates were potentially involved in neurological and psychiatric processes, warranting further investigation into their potential role in the biological mechanisms of depression. These results provide preliminary insights and should be considered exploratory and hypothesis‐generating, warranting validation in future studies with larger and independent cohorts.

Major depressive disorder is a prevalent and debilitating mental disorder that affects millions of individuals worldwide, imposing a substantial burden on both individuals and society.^1^ The underlying molecular mechanisms and biomarkers associated with depression, including the full scope of depressive disorders and depressive symptoms, remain poorly understood. Recent advances in molecular biology have shed light on microRNAs (miRNAs), small non‐coding RNA molecules that play crucial roles in the post‐transcriptional regulation of gene expression. MiRNAs can cross the blood–brain barrier, allowing them to serve as potential indicators of neural processes associated with mental disorders.^2^ MiRNAs may therefore act as promising candidates for understanding the pathophysiology of depression or as potential biomarkers, not only due to their involvement in neural processes,^3^, ^4^, ^5^ but also because of their detectability in peripheral biological fluids, like plasma.

Previous studies have identified a variety of miRNAs as potential biomarkers for depression, with findings indicating both upregulated and downregulated miRNAs in depressed individuals compared to healthy controls. A systematic review of circulatory miRNAs in major depressive disorder reported that 20 circulating miRNAs have been identified in two independent studies, and 10 in three or more. Most of these miRNAs are highly expressed in brain tissues and regulate neuronal and major depressive disorder associated processes.^6^ For example, upregulation of miR‐132 serves as an important regulator of neurotropic action, mainly through the activation of hippocampal BDNF‐ERK‐CREB signaling pathways.^7^ Likewise, miR‐124‐3p is expressed in the brain of individuals with major depressive disorder^8^, ^9^ and was proposed as a therapeutic target for anti‐depressants^10^ or dysregulation of the let‐7 family miRNAs has been identified as candidate biomarkers for major depressive disorder.^11^, ^12^, ^13^

Despite these promising findings, several key gaps remain to be addressed. First, many studies to date have been based on small, selected clinical samples using a case–control design.^6^ As such, little is known about how miRNA expression levels may associate with depressive symptoms in the general population.^14^ This is important given that depressive symptoms manifest along a continuum, and that individuals with subclinical scores on depression measures may still show dysregulated expression levels of circulating miRNAs. Second, studies have varied widely in the methods used for detecting miRNAs levels, mainly utilizing qRT‐PCR to measure specific candidate miRNAs, which limits comparability between findings and also means that other miRNAs relevant to depression may remain undetected, as they were not tested.^14^ Third, studies to date have primarily focused on individuals during early and mid‐adulthood. Yet, models of genetic and environmental influences on depression suggest that depression may have a heterogeneous pathophysiology over the lifespan,^15^ indicating that miRNA profiles associated with depression may also change in older age.

To address these gaps, we aimed to explore associations between plasma‐derived extracellular miRNA profiles and depressive symptoms using data from the population‐based Rotterdam Study cohort,^16^ in order to identify potential biomarkers and molecular pathways regulated by miRNAs linked to depression. To this end, we generated data from over 2700 participants using a next‐generation RNA‐sequencing platform, allowing for simultaneous, quantitative detection of 2083 miRNAs.^17^, ^18^ This enabled testing for associations with a large set of potential miRNA biomarkers, and establishing the potential functional relevance of late life depression‐associated miRNAs using a range of *in silico* studies. Though the Rotterdam Study primarily includes middle‐aged and elderly individuals of European ancestry, the generalizability to younger or more diverse populations might be limited, so studying this cohort offers valuable insights into depressive symptoms later in life. Such insights may ultimately contribute to the development of more accurate diagnostic tools and deeper understanding of the biological mechanisms underlying this complex mental disorder.

## Materials and Methods

### Study population

This study was conducted utilizing data from the Rotterdam Study, a prospective population‐based cohort in the Netherlands ongoing since 1990.^16^ Detailed information about the Rotterdam Study has been previously published and is available in the Methods in the supporting information; Data S3.

The baseline data for the current study were collected during inclusion rounds between 2002–2005 and 2017–2018. During this period, participants visited the research center for blood sampling and underwent extensive assessments. A random subset, for whom miRNA expression in plasma was determined, was included in this study. This included 1000 participants from the fourth visit of the first cohort (RS‐I‐4), 1000 participants from the second visit of the second cohort (RS‐II‐2), who visited the center between 2002 and 2005. Moreover, we included 754 participants from the first visit of the fourth cohort (RS‐IV‐1), where miRNA expression were measured from blood samples collected in 2017–2018. One participant had insufficient miRNA data quality, and 50 participants had insufficient data on depressive symptoms. Therefore, our study population consisted of 2703 participants.

The Rotterdam Study has been approved by the Medical Ethics Committee of the Erasmus MC (registration number MEC 02.1015) and by the Dutch Ministry of Health, Welfare and Sport (Population Screening Act WBO, license number 1071272‐159521‐PG). The study was conducted in accordance with the principles of the Declaration of Helsinki. The Rotterdam Study Personal Registration Data collection is filed with the Erasmus MC Data Protection Officer under registration number EMC1712001. The Rotterdam Study project persistent identifier is https://ror.org/02ac58f22. All participants provided written informed consent to participate in the study and to have their information obtained from treating physicians.^16^

### MiRNA expression profiling

Expression levels of 2083 miRNAs in plasma were analyzed using the HTG EdgeSeq miRNA Whole Transcriptome Assay developed by HTG Molecular Diagnostics. The sequencing was performed on an Illumina NextSeq 500 sequencer (Illumina). The counts per million values of miRNA expression levels were first adjusted for total reads within each sample and subsequently log2 transformed. MiRNAs with lg2 counts per million <1.0 were indicated as not expressed in the samples.

Out of the 2083 miRNAs initially profiled, 591 miRNAs were previously identified as well‐expressed in plasma and were selected for subsequent analyses.^19^ For a comprehensive description of the methods employed for miRNA expression profiling, we refer to the Methods in the supporting information; Data S3.

### Depressive symptoms

Depressive symptoms were assessed with the Center for Epidemiologic Studies Depression (CES‐D) scale, a 20‐item scale covering cover a range of emotional, cognitive, and physical symptoms.^20^, ^21^ The total score ranges between 0 and 60, where a higher score indicates more depressive symptoms.

A weighted CESD sum score was used for participants who had answered with a maximum of five questions missing, see Methods in the supporting information; Data S3. However, if participants had more than five missing questions, CES‐D scores were not calculated and assumed missing.

### Statistical analysis

We investigated the association of each miRNA with depressive symptoms scores individually. To this end, we regressed depressive symptoms on each miRNA using negative‐binomial linear models, from which we derived mean differences and their corresponding 95% confidence intervals (95% CI). We selected miRNA with *P* < 0.05 that also met a threshold for effect size with an absolute estimate greater than 0.1. We opted for a Negative Binomial Regression due to the nature of the CES‐D scores showing signs of over‐dispersion, with a greater variance than the mean, and skewness towards the lower scores, see Fig. S1.

We accounted for BMI, white blood cells, red blood cells, education, smoking, Plate number, and well positioning. For a more detailed description of the used confounders, see Model building section in the supporting information; Data S3. A Benjamini‐Hochberg (False Discovery Rate, FDR) correction was applied to adjust for multiple testing.

### Post hoc analysis of identified miRNAs and target genes

We conducted a comprehensive set of *in silico* studies to explore the potential involvement of the identified miRNAs in underlying depression disease pathways.

### Step 1: miRNA target prediction

We first utilized two databases for miRNA target prediction, namely miRTarBase^22^ and miRDB.^23^ Predicted target genes for the identified miRNAs were extracted, with consideration given to targets having a score above 80 in miRDB and being functionally approved targets in miRTarBase.

### Step 2: Functional enrichment analysis

We evaluated whether these putative target genes collectively contributed to specific biological pathways. This was carried out using the STRING^24^ database for association networks and functional enrichment analyses. It integrates data from scientific literature, experimental data, computational prediction methods, and public databases to facilitate the analysis of interaction networks and biological processes. In our approach, we specifically modified the default settings for active interaction sources by selecting only experiments, databases, and gene fusion as our evidence types.

### Step 3: Brain expression examination

We looked up the expression of identified miRNAs in the brain. The objective was to elucidate potential links between plasma miRNA profiles and miRNAs expressed within the brain, specifically focusing on their relevance to depressive symptoms. We accessed brain expression data for miRNAs, which provided information on miRNA expression patterns within various brain regions, from the miRNATissueAtlas2.^25^

### Step 4: SNP analysis

We investigated whether single nucleotide polymorphisms (SNPs) are located within the corresponding sequences of identified miRNAs or their putative target genes, and then looked up their associations with depression. To achieve this, we made use of the genome‐wide summary statistics obtained from a prior meta‐analysis of PGC^26^ and UK Biobank^27^ focused on depression.^28^ We employed the Bonferroni correction to adjust our test statistics for the total number of miRNA and target genes combined.

### Step 5: Gene based tests

We hypothesized that target genes of the top‐associated miRNAs are more likely to be associated with depression than non‐target genes. Specifically, we tested whether target gene sets are enriched for association with depression based on GWAS summary statistics of major depressive disorder.^28^ These subsets of genes are defined as the subset of target genes of upregulated miRNAs (1), target genes of downregulated miRNAs (2), or the combination of both subsets (3). We utilized MAGMA, a tool for gene‐set analysis of GWAS summary data, which performs enrichment analyses using two steps. First, the joint effect of all SNPs within a gene is computed providing a gene‐based *P*‐value. Then, a multiple regression model is used to examine enrichment testing while accounting for linkage disequilibrium.^29^, ^30^

### Step 6: Functional mapping and annotation

Finally, we utilized FUMA^30^ to annotate the top‐associated miRNA target genes to a biological pathway. For both up‐ and down‐regulated target genes, we examined enrichment by tissue type through studying overrepresentation in sets of differentially expressed genes for 30 general tissue types and 53 tissue types as based on tissue‐specific expression patterns derived from GTEx RNA‐seq v8.^31^ This results in an overview of biological functions of up‐ and downregulated target genes.

## Results

### Participant characteristics

An overview of baseline participant characteristics and their relationship with depressive symptoms are shown in Table 1. Female participants, on average, had stronger depressive symptoms compared to males.

**Table 1:** Demographic characteristics and distribution of depressive symptoms (CES‐D scale) for the study population

Characteristics	Count	Mean (SD)	Median [25th–75th]
Age^†^			
<60	1158	6.62 (8.18)	4 [1–9]
60–70	1065	5.86 (7.39)	3 [1–8]
70–80	398	5.43 (6.66)	3 [1–7]
>80	82	5.7 (7.78)	4 [1–7]
Sex, n (%)			
Male	1160	5.55 (7.09)	3 [1–7]
Female	1543	6.62 (8.18)	4 [1–9]
Education			
Primary school	268	6.85 (9.01)	4 [1–9]
Lower	1021	6.10 (7.65)	3 [1–8]
Intermediate	879	6.19 (7.86)	3 [1–8]
Higher	535	5.87 (7.04)	4 [1–8]
Smoking			
Never	923	5.90 (7.13)	4 [1–8]
Former	1398	6.03 (7.51)	3 [1–8]
Current	382	7.27 (9.72)	4 [1–9]
BMI			
Below 18.5	17	9.76 (10.28)	5 [1–17]
18.5–24.9	1237	5.86 (7.43)	3 [1–8]
25.0–29.9	696	6.24 (7.71)	3 [1–8]
30.0 and Above	50	8.32 (9.94)	4 [1–12]

The data are categorized by age, sex, education, and smoking status, and BMI. The count, mean (standard deviation), and median (the 25th–75th percentile range) for each category are reported.

^†^ Age is in years.

### Differential miRNAs associated with depressive symptoms

**Fig. 1:**
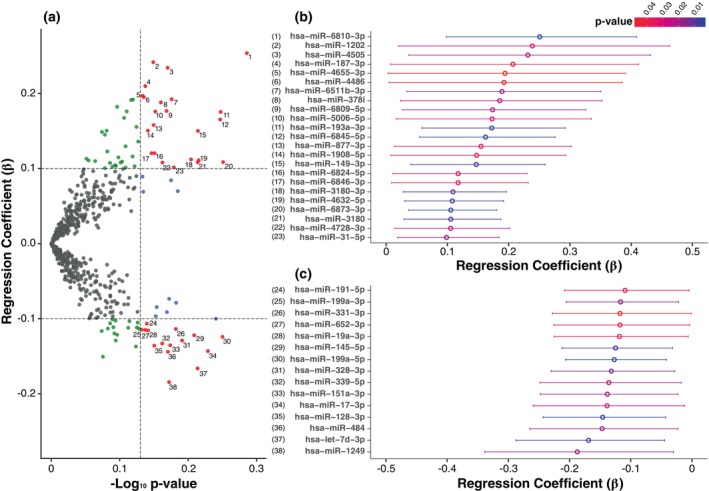
Association of miRNAs with depressive symptoms. Panel (a) presents a volcano plot displaying the relationship between miRNAs and depressive symptom (CES‐D) scores, highlighting miRNAs with significant *P*‐values (y‐axis) and regression coefficients (x‐axis). Panels (b) and (c) show forest plots of the top upregulated (b) and downregulated (c) miRNAs with corresponding 95% confidence intervals.

We employed fully adjusted negative‐binomial linear regression models to investigate the association between the expression levels of 591 miRNAs and the depressive symptoms scores (Fig. 1a, Supplementary Material 1). Our exploratory analysis identified 38 miRNAs showing nominal associations with depressive symptoms (*P*‐value <0.05 and absolute effect estimate > = 0.1). For an estimate of 0.1, a one‐unit increase in the log‐transformed expression is associated with a 10.5% increase in the expected score on depressive symptoms, holding other variables constant. Of note, none of these associations remained significant after applying FDR multiple testing correction. The top associated miRNA was miR‐6810‐3p (*P*‐value~0.001, beta = 0.25, SE = 0.08). Among the 38 nominally associated miRNAs, 23 were found to be upregulated (Fig. 1b) and 15 miRNAs displayed downregulated expression (Fig. 1c) in participants with higher scores on depressive symptoms.

We next conducted some additional downstream analyses in order to assess the potential functional significance of the 38 identified miRNAs in depression by delving into their brain expression, putative target genes and relevant pathways. Despite our lack of miRNAs passing FDR correction; exploring the downstream enrichment, tissue‐specific expression, and genetic overlap of these miRNAs may still prove informative. Taking the overlap in results between target genes from miRDB and miRTarBase led to the identification of 456 unique putative target genes, for which 241 were targets of upregulated miRNAs (Fig. S2) and 246 were targets of downregulated miRNAs (Fig. S3). The enrichment *P*‐values for the targets of upregulated miRNAs and downregulated miRNAs, compared to the expected number of gene interactions at random versus the observed interactions in our results, are 2.33e^−10^ and <1.0e^−16^, respectively. A full list of the 38 miRNAs and their target genes is added as Supplementary Material 2.

### Enrichment analysis

To further investigate the retrieved miRNA target genes, we proceeded with a functional enrichment analysis using STRING.^24^ This analysis was performed separately for the targets of up‐ and down‐regulated miRNAs. Notably, both showed significant enrichment of head, brain, and (central) nervous system pathways. For the targets of upregulated miRNA, key associations emerged with terms like ‘Neuron to neuron synapse’ and ‘Postsynaptic density’, as shown in Table 2a. The targets of downregulated miRNA on the other hand provide terms as ‘Negative regulation of neurotransmitter secretion’ and pathways related to EGFR and TGF‐B, as shown in Table 2b.

**Table 2:** This table lists the pathways targeted by upregulated miRNAs

Database	Description	Count in network	Strength	FDR
a. For 23 upregulated miRNAs				
Cellular Components (Gene Ontology)	Neuron to neuron synapse	18 of 355	0.63	0.0011
	Postsynaptic density	15 of 324	0.59	0.0056
	Anchoring junction	36 of 1325	0.36	0.0032
	Synapse	36 of 1350	0.35	0.0036
Tissue expression	Brain	108 of 5733	0.2	0.0001
	Nervous System	112 of 6016	0.19	0.0001
	Central Nervous System	109 of 5825	0.19	0.0001
	Head	114 of 6642	0.16	0.0012
WikiPaths (UniProt)	ErbB signaling pathway	8 of 89	0.88	0.0156
b. For 15 downregulated miRNAs				
Biological Process (Gene Ontology)	ERBB2‐ERBB4 signaling pathway	3 of 5	1.71	0.0071
	Negative regulation of neurotransmitter secretion	3 of 11	1.36	0.0308
	Epidermal growth factor receptor signaling pathway	6 of 49	1.02	0.0041
Molecular Functioning (Gene Ontology)	SMAD binding	7 of 78	0.88	0.0370
Tissue Expression	Brain	121 of 5733	0.25	8.51e‐10
	Nervous system	123 of 6016	0.24	1.16e‐09
	Head	126 of 6642	0.21	6.05e‐08

The ‘Count in Network’ column shows the number of miRNAs involved in each pathway out of the total considered, while ‘Strength’ indicates the pathway's relevance to the network of upregulated miRNAs. The ‘False Discovery Rate (FDR)’ provides the adjusted *P*‐value, indicating the significance of the pathway's association with the upregulated miRNAs.

### Brain expression

**Fig. 2:**
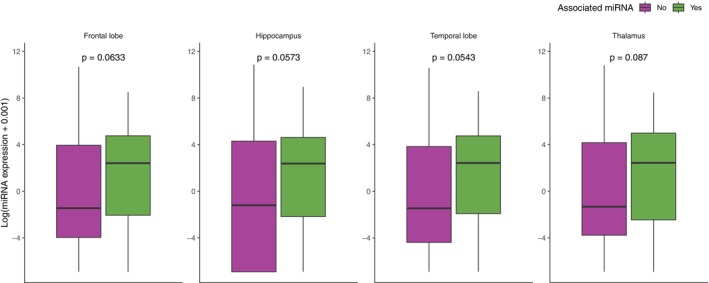
Box plots illustrating the differential expression of 38 miRNAs associated with depressive symptoms across various brain regions. The comparison is between cases where the miRNA is associated with a high CES‐D score (green) and non‐associated cases (purple). The expression levels are log‐transformed for normalization. Statistical significance is indicated by *P*‐values from a Wilcoxon Signed Rank test.

We looked up the miRNATissueAtlas2^25^ to explore the expression patterns of 38 top‐ranking miRNAs. We aimed to ascertain if these miRNAs displayed higher expression in the brain regions commonly affected in depression, such as the frontal lobe and hippocampus. MiRNAs nominally associated with depression displayed heightened expression in the brain, but the difference to non‐associated miRNA was not statistically significant, albeit suggestive (*P* = 0.054) (Fig. 2).

### Genetic association study

We identified common SNPs located within the sequences of 38 miRNAs and their target genes, as described in the methods section. We then investigated these SNPs for significant associations with depression using previous genetic data.^28^ Given the number of tests we applied a significance threshold, adjusting for multiple testing with a corrected *P*‐value of <0.0001.

**Fig. 3:**
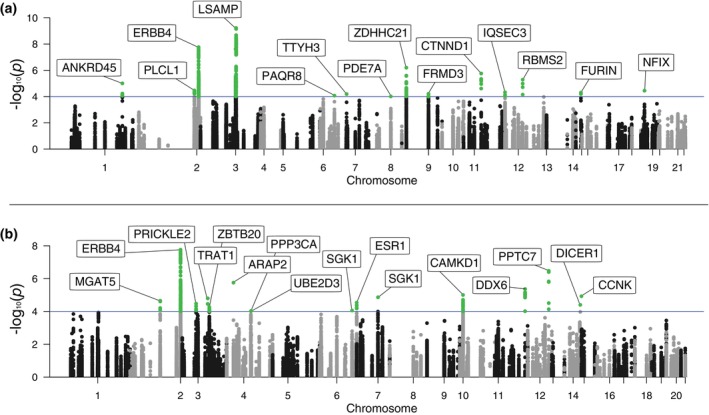
Genomic Distribution of SNPs in miRNA Target Genes Associated with depressive symptoms. Panel (a) displays a Manhattan plot illustrating the significance of SNPs within genes targeted by upregulated miRNAs across chromosomes, with highlighted genes surpassing the threshold for suggestive significance. Panel (b) shows a similar distribution for SNPs in genes targeted by downregulated miRNAs, with key genes annotated above the line indicating the level of statistical significance against the backdrop of the genomic landscape. The genetic data presented in this figure were obtained from a combined GWAS conducted by Howard *et al*. in 2019.

Five of the SNPs present in the summary statistics of the GWAS^28^ were found to be within the pre‐miRNA base pairs of the identified miRNAs, but none of them were significantly associated. Further investigation revealed that 765 significantly associated unique SNPs (*P* < 0.0001) were located within the miRNA target genes Fig. 3. Of those, 682 were in the 14 gene targets of upregulated miRNAs (Fig. 3a) and 454 were in 16 gene targets of downregulated miRNAs (Fig. 3b). *ERBB4* was found to be a target of both upregulated and downregulated miRNAs.

### Gene based tests

We tested whether any of our three subsets of target genes (all, up‐, and down‐regulated) were enriched for association with depression based on GWAS summary statistics. For the target genes of upregulated miRNAs, this failed to yield a significant result (*P* = 0.177), however, the selection targets of both up‐ and downregulated miRNAs (*P* = 0.045) as well as the selection of exclusively targets of downregulated miRNAs (*P* = 0.031) did show significantly enriched association statistics. This suggests that the positive finding for the combined set may be driven especially by the target genes of downregulated miRNAs. When we consider the selection of genes in our targets of downregulated miRNAs, the gene with the strongest association with depression was *ERBB4* (*P* = 1.18 × 10^−^^12^). This finding suggests that not only *ERBB4* is involved in depression when examining on a SNP‐level, as per our finding in the previous section, it is involved on a gene‐level as well.

### Functional mapping and annotation

We examined enrichment by tissue type through overrepresentation in pre‐defined sets of differentially expressed genes for 30 general tissue types and 53 tissue types derived from GTEx RNA‐seq v8.^31^ Notably we found that general brain tissue (Fig. S4) as well as specific brain areas (Fig. S5) including the nucleus accumbens, caudate nucleus, amygdala, cingulate cortex BA24, frontal cortex BA9, basal ganglia, hippocampus, hypothalamus, substantia nigra, and brain cortex were differentially expressed in our upregulated target genes. With the exception of the brain cortex, all these brain areas were both up‐ and downregulated themselves by our set of upregulated target genes. This may indicate that target genes of the miRNAs that were positively associated with depression were found to have a mixed effect on regulation in these brain areas. For the target genes of negatively associated miRNAs, we found the general brain tissue to be downregulated by these genes (Fig. S6). This effect persisted when performing two‐sided testing but not when testing for upregulation, suggesting a dilution of the signal in the two‐sided test relative to the test for downregulation. Specific brain areas that showed downregulation by the target genes of our negatively associated miRNAs were the amygdala, putamen, caudate nucleus, brain cortex, hippocampus, anterior cingulate cortex BA24, substantia nigra, nucleus accumbens, frontal cortex BA9, hypothalamus, cerebellar hemisphere, and the cerebellum (Fig. S7). For these specific brain areas, the target genes of our negatively associated miRNAs showed a downregulated effect. Only the cervical spinal cord showed significance in both up‐ and downregulated differentially expressed genes, suggesting a mixed signal deriving from the set of target genes of our negatively associated miRNAs.

## Discussion

In this study, we examined plasma circulatory miRNAs profiles associated with depressive symptoms based on cohort data from over 2700 individuals from the general elderly population. We identified 38 miRNAs that were suggestively associated with depressive symptoms, including 23 upregulated and 15 downregulated miRNAs. Although these associations did not survive multiple testing correction, our down‐stream *in silico* analyses of the indicative miRNAs and their putative target genes revealed enrichment for relevant molecular pathways, supporting their potential role in the pathophysiology of depression. These results provide preliminary insights, are exploratory and hypothesis‐generating in nature, and warrant validation in future studies with larger and independent cohorts.

Several of the identified miRNAs in this study are in line with existing literature suggesting a role of these miRNAs in elderly age‐related neural processes and mental disorders.^3^, ^4^, ^5^ Among the suggestive miRNAs identified as upregulated, miR‐6810‐3p is implicated in counteracting age‐related macular degeneration and neurodegenerative processes.^32^ Moreover, miR‐1202 has been shown to be upregulated in post‐mortem brain tissue of people with Alzheimer's disease.^33^ MiR‐4505 expression in peripheral blood mononuclear cells was also negatively correlated with symptom severity in patients with general anxiety disorder.^34^ Furthermore, the 3p strand of miR‐4505 is linked to Parkinson's disease, which plays a role in early neurodegeneration,^35^ a possible comorbidity suggesting a heterogeneous pathophysiology of depression over the lifespan. MiR‐187‐3p has been linked to anxiety disorders through downregulation after contextual fear conditioning in mice, modulating contextual fear memory in the hippocampus.^36^ Geaghan *et al*. found an enrichment of miRNA binding site variants among miR‐31‐5p targets in individuals with major depressive disorder, supporting its potential role in depression.^37^ Elevated plasma levels of miR‐193a‐3p were found in schizophrenia patients. Given the high prevalence of depressive symptoms in schizophrenia, this finding may suggest a role of miR‐193a‐3p in mood regulation and warrants further investigation into the potential for shared molecular mechanisms across psychiatric disorders.^38^

In the downregulated miRNAs group, miR‐1249's anti‐inflammatory properties suggest broader implications in stress and depression‐related inflammation.^39^ Let‐7d has been associated with multiple psychiatric disorders, regulating dopamine D3 receptors and influencing neuropsychiatric pathways and showed improvement of depression‐ and anxiety‐like behavior in mice.^40^ MiR‐484 in plasma has been found to be downregulated in relation to bipolar disorder.^41^ Furthermore, downregulation of miR‐484 has been reported in association with late‐life depression and elevated risk for dementia through synaptic plasticity, synaptic transmission, and mitochondrial fission.^42^ Higher levels of miR‐652‐3p related to higher probabilities of bipolar disease when comparing euthymic bipolar patients under lithium treatment and healthy controls.^43^ MiR‐128‐3p, implicated in Alzheimer's disease, suppresses tau phosphorylation and reduces amyloid‐beta accumulation, potentially positioning it as a significant player in neurodegenerative processes.^44^ Additionally, *ERBB4* was found to be one of the target genes of the downregulated group and contains rs11693031, which is related to depression.^45^ Other than depression, ERBB4 was found to be related to Alzheimer's disease,^46^ loneliness day,^47^, ^48^ and household income.^49^

Among our findings were negative associations between depression and miRNAs identified in prior studies, such as let‐7d‐3p. This miRNA was identified in the context of depression before, although the direction of effect does not seem to be consistent with our findings. Let‐7d‐3p was found upregulated for major depressive disorder in both CSF (*n* = 12) and serum (*n* = 53), albeit in smaller sample sizes than the current study.^50^ Although there is a chance of a false positive finding in either previous or current studies, this observation may also reflect an effect depending on study contexts such as age and population examined. For example, mean ages ranged between 32 years for cases and controls in CSF‐samples, and 34 years in serum‐samples, whereas our sample's mean age was 62 years.

Overall, our findings are suggestive and should therefore be interpreted with caution. Nevertheless, the identified miRNAs may represent interesting targets for future research and if replicated, could help in designing novel diagnostic tools. Currently, the diagnosis of depression relies heavily on clinical assessment, which is subjective.^51^, ^52^ The identified miRNAs in our study may represent candidate biomarkers which may eventually contribute to more precise and early diagnosis. Furthermore, understanding the target genes and pathways regulated by these miRNAs could open new avenues for therapeutic interventions, potentially leading to the development of miRNA‐based therapies.

Our study has several strengths, which are outlined here. First, we leverage a large cohort, enhancing the statistical power and representativeness of findings relative to a middle‐aged and elderly population. Second, our use of a targeted RNA‐sequencing based method ensures high sensitivity and reproducibility, addressing limitations related to methodological inconsistencies in measurement noted in earlier research and covering a large number of human miRNAs.^6^, ^14^ Third, we adjusted rigorously for multiple testing, which has been unevenly applied in many existing studies on miRNAs and behavioral outcomes. Additionally, as a population‐based study, our work encompasses a broad, elderly demographic, extending our understanding of the relationship between miRNAs and dimensional depressive symptoms at an older age. Our study is also not without limitations. The cross‐sectional and observational nature of our analysis limits our ability to infer causality. Longitudinal studies are needed to determine whether changes in plasma miRNA expression precede the onset of depressive symptoms or are a consequence of the disorder. Furthermore, while our sample size was relatively large compared to previous studies, it may still have limited power to detect small effect sizes after correction for multiple testing. This limitation could have contributed to the absence of significant findings after multiple testing correction. Based on our sample size, detectable associations are likely limited to miRNAs with moderate to large effect sizes. Mendelian Randomization may help assess potential causal links between identified miRNAs and depressive symptoms, strengthening biological interpretation beyond observational findings in future studies. However, this approach requires strong and valid genetic instruments for the miRNAs, which are currently lacking. Additionally, our study population is an elderly population of predominantly European descent, which may limit the generalizability of our findings to other age cohorts, such as adolescents, or to different ethnicities. Future studies should include more ethnically diverse populations to enhance the generalizability of the results. Finally, we highlighted target genes and pathways for suggestive miRNAs in our downstream analyses. These downstream analyses were based on nominally significant results, and permutation‐based validation was not feasible due to limitations of the STRING tool, which restricts large‐scale automated querying. Exploring the functional roles of these miRNAs in gene regulation in relevant cell or animal models could provide direct evidence of their involvement in depression‐related pathways. Investigating interactions between these miRNAs and environmental factors, such as stress or trauma, could offer insights into the etiology of depression.

Collectively, we examined plasma circulatory miRNA profiles associated with depressive symptoms based on data from the general elderly population. Our findings suggest associations between several circulating miRNAs and depressive symptoms. Our follow‐up *in silico* analyses highlight that miRNAs with the largest effect estimates did appear to be involved in important neurological and psychiatric processes, suggesting a possible role in the biological mechanisms of depression that warrant further validation.

## Disclosure statement

The authors declare no conflict of interest.

## Funding Information

The Rotterdam Study is supported by the Erasmus Medical Center and the Erasmus University Rotterdam, the Netherlands Organization for Scientific Research (NWO), the Netherlands Organization for Health Research and Development (ZonMw), the Research Institute for Diseases in the Elderly (RIDE), the Ministry of Education, Culture, and Science, the Ministry of Health, Welfare and Sports, the European Commission (DG XII), and the municipality of Rotterdam. MiRNA expression profiling was funded by the Janssen Prevention Center of Janssen Vaccines and Prevention BV, part of the Janssen Pharmaceutical Companies of Johnson & Johnson. The work of MK and MG was partly supported by the Erasmus MC Fellowship (EMCF20213) and Alzheimer Nederland (WE.03‐2021‐10) grants. The work of TF, AN and CAMC was supported the European Union's Horizon Europe Research and Innovation Programme (FAMILY, grant agreement No 101057529). AN and CAMC are supported by the European Research Council (TEMPO; grant agreement No 101039672). The work of CAMC is further supported by the European Union's Horizon 2020 Research and Innovation. Programme (EarlyCause, grant agreement No 848158). This research was conducted while CAMC was a Hevolution/AFAR New Investigator Awardee in Aging Biology and Geroscience Research. The mentioned funders had no role in the design and conduct of the study, nor in the decision to submit the manuscript for publication.

## Author contributions

Conceptualization: MG. Data curation: MG, AIL. Formal analysis: MMK, TF. Funding acquisition: MG, CAMC. Investigation: MG, AIL. Methodology: MG, AN, CAMC. Project administration: MG. Resources: MG, AIL, MAI. Software: MMK, TF. Supervision: MG, AN, CAMC. Validation: MG, AN, CAMC. Visualization: MMK, TF. Writing – original draft: MMK, TF. Writing – review and editing: All authors.

## Supporting information


**Data S1.** Supporting information.

**Data S2.** Supporting information.

**Data S3.** Supporting information.

## Data Availability

The Rotterdam Study data can be made available to interested researchers upon request. Requests can be directed to data manager Frank J. A. van Rooij (f.vanrooij@erasmusmc.nl) or visit the following website for more information: https://www.ergo-onderzoek.nl/contact. We are unable to place data in a public repository due to the confidential nature of the data collected and legal and ethical constraints. The summary of the results from our miRNA projects are available through https://www.mirnomics.com/.
