# Supplementary material for: Plasma circulating microRNAs and symptoms of depression: Results from a population‐based study
Source: Psychiatry Clin Neurosci. 2025 Jul 14;79(10):636–44. doi: 10.1111/pcn.13869 (PMC12498121; doi:10.1111/pcn.13869)
Supplement: Supplementary file 3 — Data S3. Supporting information. [file PCN-79-636-s001.docx]

Supporting methods for "Plasma circulating microRNAs and symptoms of depression: Results from a population-based study"

**Supplementary Methods 1: Study Design and Data Collection**

This study uses data from the Rotterdam Study, focusing on individuals assessed for depressive symptoms and for whom microRNA (miRNA) expression data was measured. The data includes psychiatric evaluations, blood samples for miRNA analysis, and relevant demographic and health information. The Rotterdam Study began its first phase, RS-I, in 1990 by inviting all residents aged 55 years and above from Ommoord, a Rotterdam suburb, to participate. Out of those invited, 7,983 individuals consented to join the study. In the year 2000, the second wave, RS-II, applied the same inclusion criteria and successfully enrolled 3,011 individuals. The third wave, RS-III, took place in 2006 and extended invitations to individuals aged 45 years and above, resulting in 3,932 participants. This study continues to this day, with follow-up examinations conducted every 3-5 years. For a more detailed overview of the study, please refer to our previously published works [1].

**Supplementary Methods 2: miRNA Expression Profiling**

To conduct miRNA expression profiling, blood samples were collected and processed in EDTA-treated containers, plasma was then aliquoted and stored at -80°C. The HTG EdgeSeq miRNA Whole Transcriptome Assay (WTA) from HTG Molecular Diagnostics (Tucson, AZ, USA) and the Illumina NextSeq 500 sequencer (Illumina, San Diego, CA, USA) were utilized to measure cell-free miRNA expression levels in plasma. This assay covered 2,083 mature human miRNAs, including 13 housekeeping genes, which were crucial for data normalization and subsequent analysis. For normalization purposes and to adjust for total reads per sample, miRNA expression levels were converted into counts per million (CPM) and Log2 transformed. We set a threshold of 1.0 Log2 CPM, classifying values below this cutoff as insufficiently expressed. To define well-expressed miRNAs in our sample, we utilized a monotonic decreasing spline curve between the means and standard deviations of all miRNAs to determine the lower limit of quantification (LLOQ). According to this criterion, miRNAs with at least 50% above the LLOQ were considered well-expressed, resulting in the identification of 591 well-expressed miRNAs out of the total set of 2,083 miRNAs. These well-expressed miRNAs were used for subsequent analyses.

**Supplementary Methods 3: CES-D measurement**

Symptoms of depression were quantified using the Center for Epidemiologic Studies Depression Scale (CES-D). Participants' scores were used to evaluate the severity of depressive symptoms. The CES-D scale is a self-report questionnaire comprising 20 items that assess the frequency of depressive symptoms experienced in the past week. Each item is rated on a 4-point scale ranging from 0 ("rarely or none of the time") to 3 ("most or all of the time"). The total score ranges from 0 to 60, with higher scores indicating greater depressive symptomatology. The scale includes questions targeting various domains of depression, such as mood, appetite, sleep disturbances, and feelings of guilt and worthlessness. CES-D scores were treated as continuous variables in statistical analyses to capture the full spectrum of depressive symptom severity. For analyses aiming to categorize depression status, cut-off points were applied as specified above to differentiate between participants with and without clinically significant depressive symptoms.

[1] Ikram MA, Kieboom BCT, Brouwer WP, et al. The Rotterdam Study. Design update and major findings between 2020 and 2024. Eur J Epidemiol. 2024;39(2):183-206. doi:10.1007/s10654-023-01094-1

**Model building**

The skewed distribution and high frequency of zero values of CES-D scores made standard linear models unsuitable. The negative binomial model more accurately captured these characteristics, ensuring a better fit compared to other approaches.

We controlled for a set of confounders that are known or suspected to influence both miRNA expression and depressive symptoms. These include:

- **Age**: Age is a critical confounder as both miRNA expression patterns and depressive symptoms can vary significantly with age.
- **Cohort**: The cohort variable accounts for potential differences between the three ERGO that may influence the relationship between miRNA levels and depressive symptoms.
- **BMI**: Obesity and metabolic health are linked to both altered miRNA profiles and mood disorders, including depression.
- **White Blood Cell and Red Blood Cell counts**: These blood parameters can influence miRNA expression and are also associated with inflammation, a known factor in depression.
- **Education Level**: Education is often used as a proxy for socioeconomic status, which is a known determinant of both mental health outcomes and biological factors influencing miRNA.
- **Smoking Status**: Smoking has been shown to affect both miRNA expression and mental health, thus making it a necessary confounder.
- **Plate Number and Well**: These variables control for potential technical variation due to the experimental setup (batch effects), ensuring that any associations we observe between miRNA levels and depressive symptoms are not driven by artifacts in the measurement process.

**Supplemental figure S1: CESD distribution**

Figure 1: This figure provides overview of the distribution of depressive symptomatology, as quantified by the Center for Epidemiologic Studies Depression Scale (CESD). The right-skewed histogram represents individual CESD scores. Statistical annotations highlight a mean score, a median, a standard deviation (SD), and interquartile range.

**Supplemental figure S2: Targets of Upregulated miRNA**


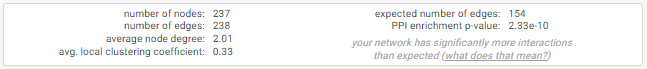


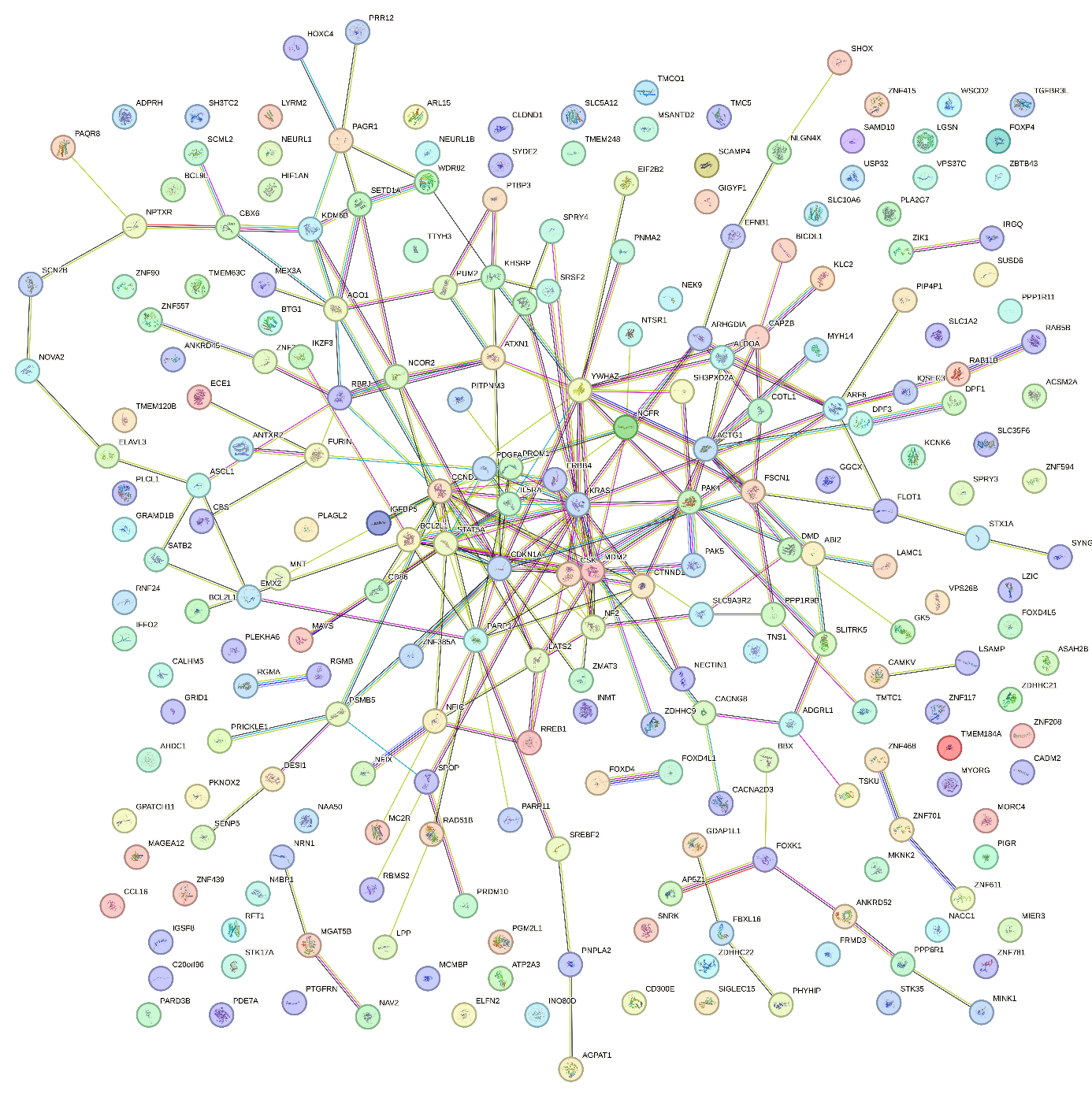


Network Visualization of Upregulated miRNA Gene Targets: This diagram illustrates the complex interactions between genes that are regulated by a set of upregulated miRNAs.

**Supplemental Figure S3: Targets of Downregulated miRNA**


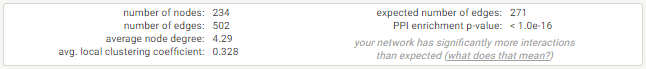


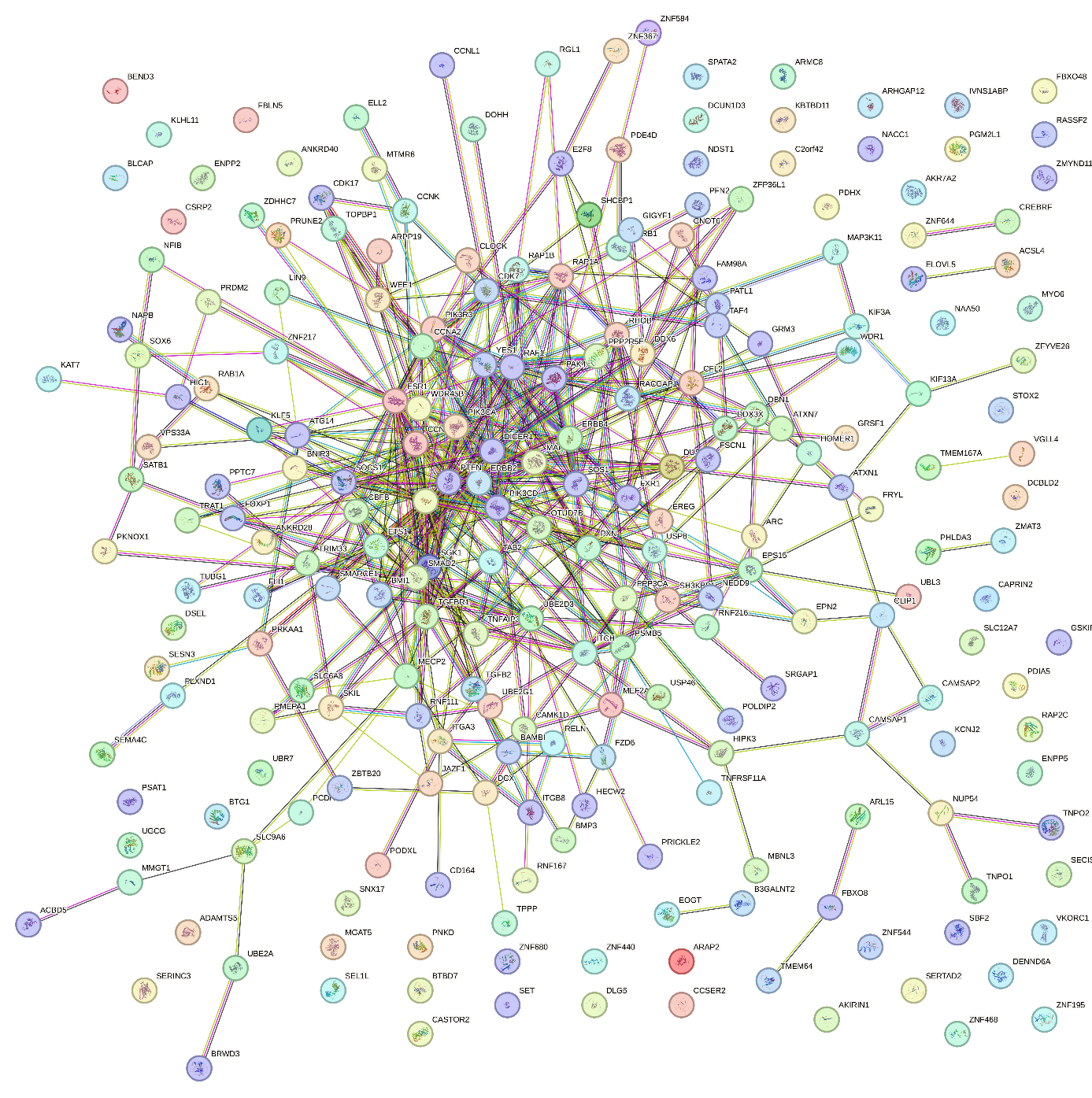


Network Visualization of Upregulated miRNA Gene Targets: This diagram illustrates the complex interactions between genes that are regulated by a set of downregulated miRNAs.

**Supplemental Figure S4. General tissue specificity. Positively associated miRNA target genes’ differential expression in general tissues.**


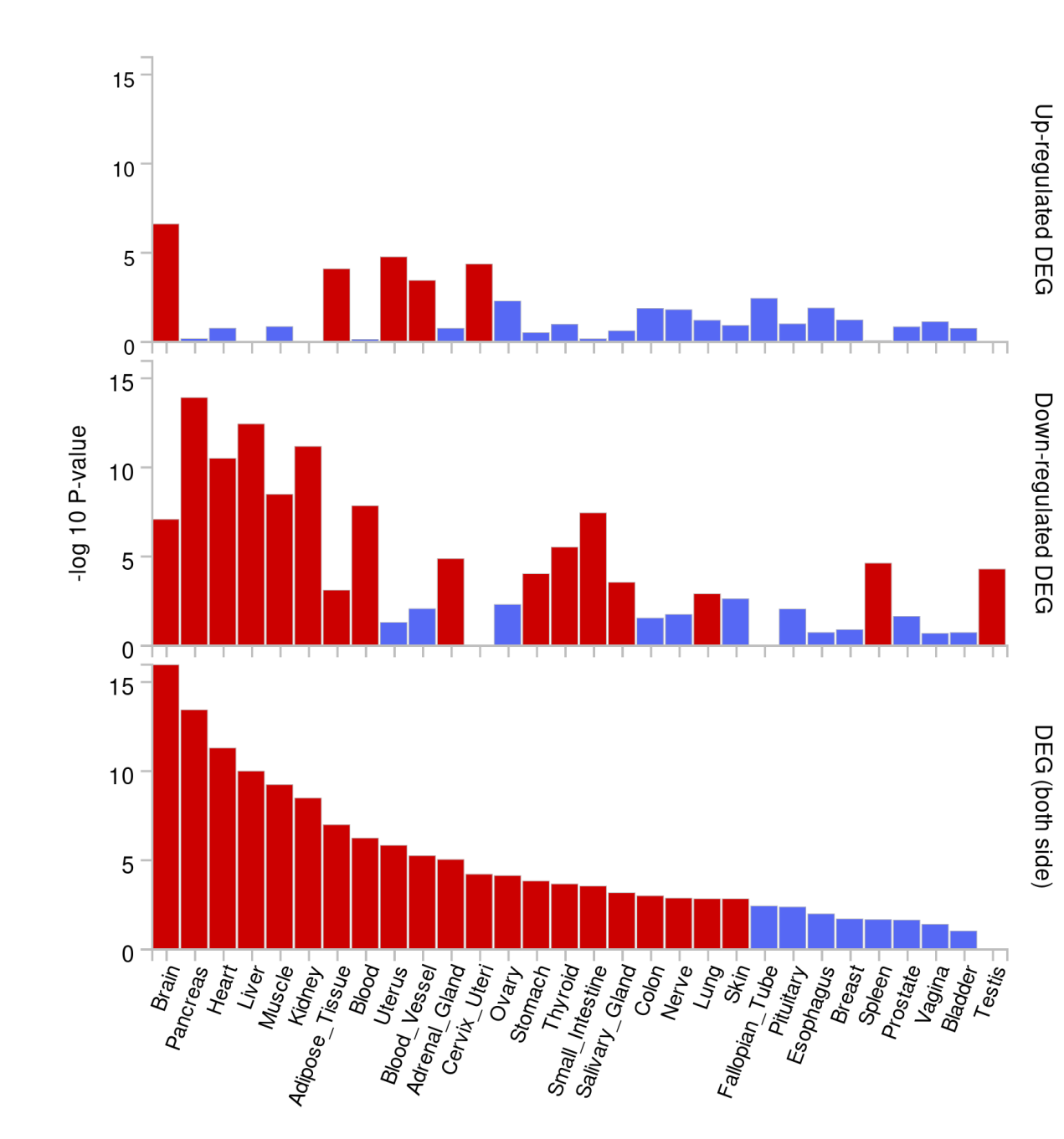


General tissue specificity for target genes of miRNAs positively associated with depression derived using FUMA. The figure portrays testing of target genes for upregulated effects, downregulated effects and two-sided t-testing on general tissue types. Statistical significance (Bonferroni corrected) is indicated by red coloring.

**Supplemental figure S5. Tissue specificity. Positively associated miRNA target genes’ differential expression in general tissues.**


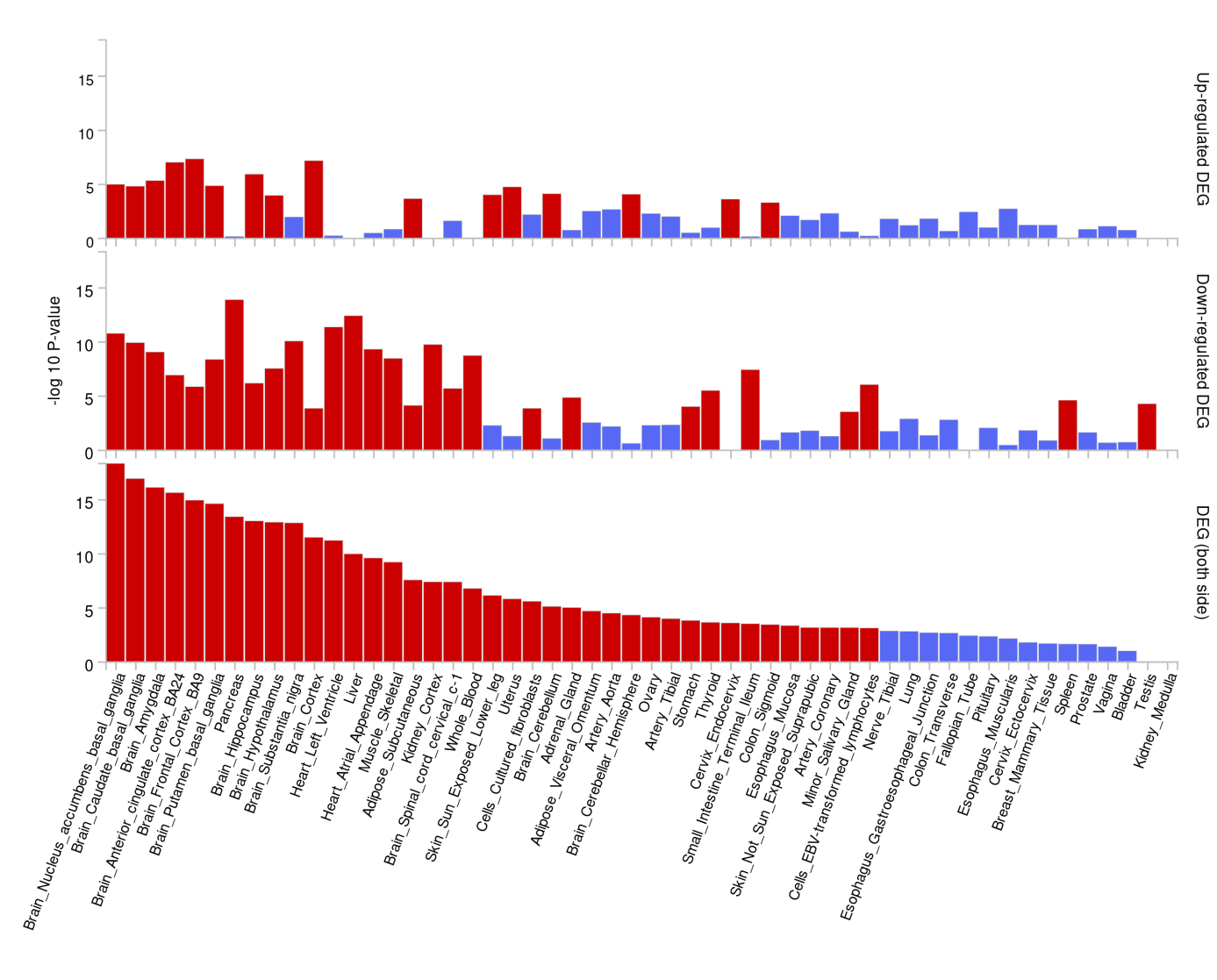


Tissue specificity for target genes of miRNAs positively associated with depression derived using FUMA. The figure portrays testing of target genes for upregulated effects, downregulated effects and two-sided t-testing on specific tissue types. Statistical significance (Bonferroni corrected) is indicated by red coloring.

**Supplemental Figure S6. General tissue specificity. Negatively associated miRNA target genes’ differential expression in general tissues.**


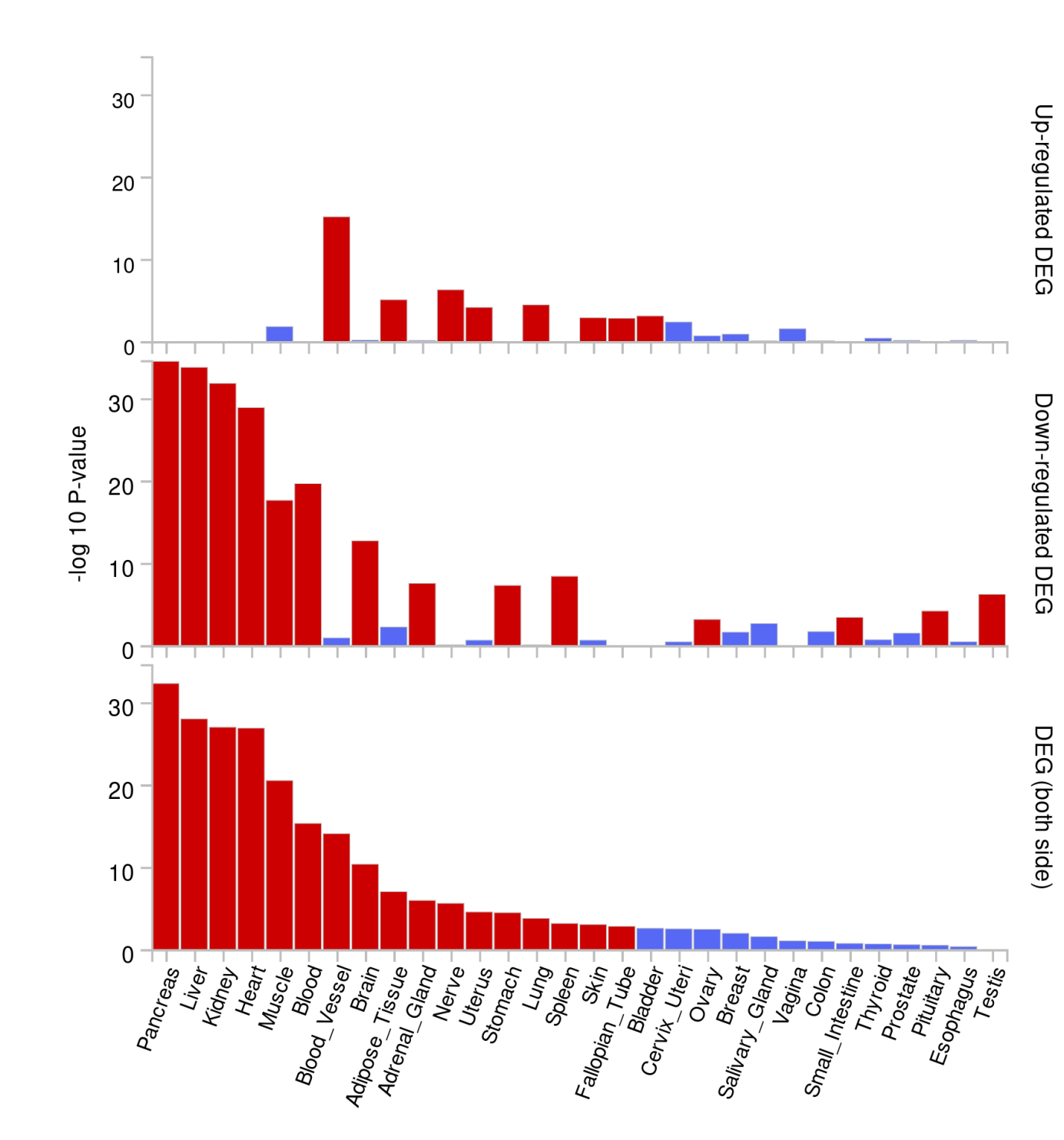


General tissue specificity for target genes of miRNAs negatively associated with depression derived using FUMA. The figure portrays testing of target genes for upregulated effects, downregulated effects and two-sided t-testing on general tissue types. Statistical significance (Bonferroni corrected) is indicated by red coloring.

**Supplemental Figure S7. Tissue specificity. Negatively associated miRNA target genes’ differential expression in general tissues.**


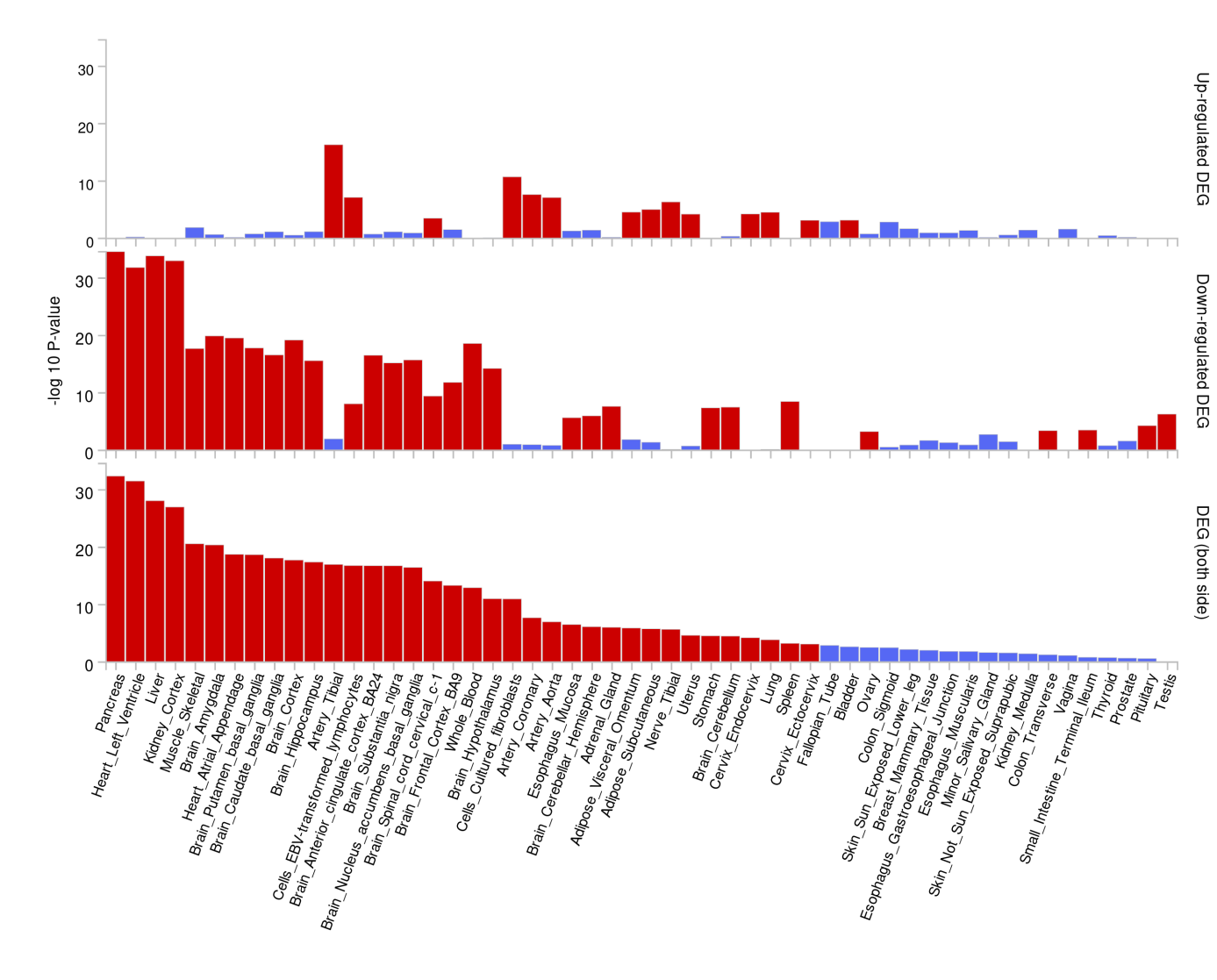


Tissue specificity for target genes of miRNAs negatively associated with depression derived using FUMA. The figure portrays testing of target genes for upregulated effects, downregulated effects and two-sided t-testing on specific tissue types. Statistical significance (Bonferroni corrected) is indicated by red coloring.
